# Cloning and Characterization of Novel Testis-Specific Diacylglycerol Kinase η Splice Variants 3 and 4

**DOI:** 10.1371/journal.pone.0162997

**Published:** 2016-09-19

**Authors:** Eri Murakami, Takao Shionoya, Suguru Komenoi, Yuji Suzuki, Fumio Sakane

**Affiliations:** Department of Chemistry, Graduate School of Science, Chiba University, Chiba, Japan; Centre National de la Recherche Scientifique, FRANCE

## Abstract

Diacylglycerol kinase (DGK) phosphorylates DG to generate phosphatidic acid. Recently, we found that a new alternative splicing product of the DGKη gene, DGKη3, which lacks exon 26 encoding 31 amino acid residues, was expressed only in the secondary spermatocytes and round spermatids of the testis. In this study, we cloned the full length DGKη3 gene and confirmed the endogenous expression of its protein product. During the cloning procedure, we found a new testis-specific alternative splicing product of the DGKη gene, DGKη4, which lacks half of the catalytic domain. We examined the DGK activity and subcellular localization of DGKη3 and η4. DGKη3 had almost the same activity as DGKη1, whereas the activity of DGKη4 was not detectable. In resting NEC8 cells (human testicular germ cell tumor cell line), DGKη1, η3 and η4 were broadly distributed in the cytoplasm. When osmotically shocked, DGKη1 and η4 were distributed in punctate vesicles in the cytoplasm. In contrast, DGKη3 was partly translocated to the plasma membrane and co-localized with the actin cytoskeleton. These results suggest that DGKη3 and η4 have properties different from those of DGKη1 and that they play roles in the testis in a different manner.

## Introduction

**Fig 1:**
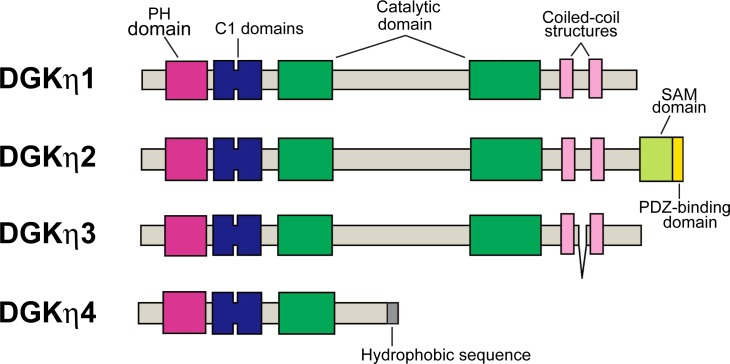
Schematic representation of the domain architectures of DGKη1, DGKη2, DGKη3 and DGKη4. DGKη3 and DGKη4 are new alternative splice variants.

Diacylglycerol kinase (DGK) phosphorylates diacylglycerol to generate phosphatidic acid [1–6]. Diacylglycerol, which is liberated from phosphatidylinositol 4,5-bisphosphste and phosphatidylcholine upon cell stimulation, regulates a wide range of cellular functions. It is well known that DGK represents a large enzyme family. Ten mammalian DGK isozymes, namely α, β, γ, δ, ε, ζ, η, Θ, ι and κ, which contain two or three characteristic cysteine-rich C1 domains and the catalytic region in common, are subdivided into five subgroups according to their structural features [1–6]. The type II DGK [7] comprises the δ [8], η [9] and κ [10] isozymes. The occurrence of alternative splicing was reported for DGKδ (δ1 and δ2) [11] and DGKη (η1 and η2 (Fig 1)) [12]. All of the type II DGK isoforms possess a pleckstrin homology domain at their N termini and a separated catalytic domain, and DGKs δ1, δ2 and η2 but not DGKs η1 or κ contain a sterile α-motif (SAM) domain at their C termini. The pleckstrin homology domain of DGKη was found to preferentially interact with phosphatidylinositol-4,5-bisphosphate [13]. Moreover, it has been reported that DGKs δ1, δ2 and η2 formed oligomers through interactions among their SAM domains and that this oligomer formation regulates the subcellular localizations of these DGK isoforms [11, 12, 14–16]. Interestingly, DGKη is a unique enzyme with high affinity for DG [17].

We recently reported that DGKη is expressed in stomach cancer and HeLa cervical cancer cells and that it is required for the Ras/B-Raf/C-Raf/MEK/ERK signaling cascade, which is activated by epidermal growth factor [18]. Moreover, DGKη was reported to be involved in lung cancer [19]. DGKη is known to be most abundantly expressed in the brain [9, 20]. Recent genome-wide association studies implicated the DGKη gene in the etiology of bipolar disorder [21–23]. Intriguingly, deficiency of DGKη indeed induced lithium-sensitive bipolar disorder (mania)-like behavior [24].

Several reports have revealed that DGKη is abundantly expressed in the reproductive organs, testis and ovary [8–12]. Therefore, we examined the expression and spatial distribution of the DGKη1 and η2 proteins and mRNAs in the mouse reproductive organs [25]. The results indicate distinct expression patterns, which were obviously different from each other. DGKη1 was distributed in the oviductal epithelium of the ovary and in the luminal epithelium of the uterus. However, DGKη2 was not detectable in reproductive organs. Moreover, we found a new splice variant of DGKη, DGKη3, which was specifically expressed in the testis. Furthermore, DGKη3 was strongly expressed in the secondary spermatocytes and round spermatids of the testis, suggesting that this isoform plays specialized roles in spermatogenesis.

In this study, we cloned the full length DGKη3 gene. Moreover, during the cloning procedure, we found a new testis-specific alternative splicing product of DGKη gene, DGKη4, which lacks the C-terminal half of DGKη3 and then examined the properties of DGKη3 and η4.

## Experimental Procedures

### Cell culture, transfection and osmotic shock

COS-7 cells were maintained in Dulbecco’s modified Eagle’s medium (Wako Pure Chemicals, Osaka, Japan) containing 10% fetal bovine serum (Corning, Corning, NY) at 37°C in an atmosphere of 5% CO_2_. cDNA was transfected into COS-7 cells by electroporation with a Gene Pulser Xcell^TM^ Electroporation System (Bio-Rad Laboratories, Tokyo, Japan) according to the manufacturer's instructions.

NEC8 (a human embryonal carcinoma cells derived from testis) were obtained from Japanese Collection of Research Bioresources (Tokyo, Japan). The cells were maintained in RPMI-1640 medium (Wako Pure Chemicals) containing 10% fetal bovine serum at 37°C in an atmosphere of 5% CO_2_. The cells were transiently transfected with cDNA using FuGENE HD Transfection Reagent (Promega, Tokyo, Japan) according to the manufacturer’s instructions. Twenty-four hours after transfection, the cells were serum starved for 3 h and incubated in RPMI-1640 with 500 mM sorbitol for 30 min.

### Plasmid constructs

cDNAs encoding for mouse DGKη1 (1–1156 aa), DGKη3 (1–1125 aa) and DGKη4 (1–558 aa) were amplified by PCR using KOD-plus DNA polymerase (Toyobo, Osaka, Japan). The products were inserted into the SalI/SacII site of pAcGFP-C1 vector (Clontech-Takara Bio, Kusatsu, Japan).

### Western blot analysis

The testes from 10-week-old male mice were homogenized in lysis buffer (50 mM HEPES, pH7.2, 150 mM NaCl, and 5 mM MgCl_2_) containing 1 mM phenylmethylsulfonyl fluoride, 20 μg/ml each of leupeptin, pepstatin, aprotinin and soybean trypsin inhibitor and Complete EDTA-free Protease Inhibitor Cocktail (Roche Diagnostics), and centrifuged at 1,000 x *g* for 5 min. The protein concentration in the supernatants was determined using a bicinchoninic acid protein assay kit (Thermo Scientific, Hudson, NH, USA). The tissue lysates (50 μg of protein) were separated on SDS-PAGE, and the separated proteins were transferred to a polyvinylidene difluoride membrane (Pall Life Sciences, Port Washington, NY, USA). The membrane was blocked with 5% skim milk and incubated with an anti-DGKη polyclonal antibody [24] overnight at 4°C. The immunoreactive bands were visualized using a peroxidase-conjugated anti-rabbit IgG antibody (Jackson ImmunoResearch Laboratories, West Grove, PA, USA) and the ECL Western Blotting Detection System (GE Healthcare Bio-Sciences, Piscataway, NJ, USA).

COS-7 cell lysates were separated on SDS-PAGE. The separated proteins were transferred to polyvinylidene difluoride membrane and blocked with 5% skim milk. The membrane was incubated with anti-GFP antibody (sc-9996, Santa Cruz Biotech, Santa Cruz, CA) for 1 h. The immunoreactive bands were visualized using peroxidase-conjugated anti-mouse IgG antibody and the Enhanced Chemiluminescence Western Blotting Detection System.

### Reverse transcription (RT)-PCR

Total RNA was isolated from each tissue of the 10 to 12-week-old male and female mice using a Direct-zol RNA MiniPrep kit (Zymo Research, Irvine, CA). cDNA synthesis was performed with the Transcription First-Strand cDNA Synthesis kit (Roche Diagnostics, Mannheim, Germany). PCR amplification was performed using rTaq polymerase (Toyobo) and the following mouse-DGKη specific primers: primer A (nucleotide positions 2416–2436, 5’-GGGAATTCCGGGAGCTACTACAGAGATC-3’) and primer B (nucleotide positions 3451–3471, 5’-CTTCCTCTGTGCCCCAATTCTG-3’). The PCR conditions for these two primers were as follows: 94°C for 3 min, 35 cycles of 94°C for 30 sec, 55°C for 30 sec, and 72°C for 2 min, and 72°C for 5 min. PCR was also performed with primer C (nucleotide positions 1411–1422, 5’- CCTGAACCTGTGGCAGCAACTG-3’) and primer D (nucleotide positions 1681–1702, 5’- GGACTCGACTGGCCTGAGAGTC-3’). The PCR conditions used for these primers were as follows: 94°C for 3 min, 35 cycles of 94°C for 30 sec, 48°C for 30 sec, 72°C for 1 min 30 sec and 72°C for 5 min.

### Confocal laser scanning microscopy

NEC8 cells grown on poly-L-lysine (Sigma-Aldrich, St. Louis, MO)-coated glass coverslips were transfected with pAcGFP-DGKη1, η3, η4 or pAcGFP vector alone. After 24 h, the cells were serum starved for 3 h and then incubated in 500 mM sorbitol for 30 min. The cells were then fixed in 3.7% formaldehyde. The filamentous actin (F-actin) was stained with Alexa 594-conjugated phalloidin (Thermo Fisher Scientific, Waltham, MA), and the nuclei were stained with 4’,6-diamino-2-phenylindole (DAPI). The coverslips were mounted using Vectashield (Vector Laboratories, Peterborough, UK). The cells were examined using inverted confocal laser microscopy (FV1000-D, Olympus, Tokyo, Japan).

### DGK activity assay

pAcGFP-DGKη1, η3, η4 or pAcGFP vector were transfected into COS-7 cells. After 48 h, the cells were harvested and suspended in ice-cold lysis buffer (50 mM HEPES (pH 7.2), 150 mM NaCl, 5 mM MgCl_2_, 1 mM dithiothreitol, complete™ EDTA-free protease inhibitor (Roche Diagnostics)) and were then sonicated. The octylglucoside mixed micellar assay of DGK activity was performed as described previously [8]. In brief, the assay mixture (50 μL) contained 50 mM MOPS (pH 7.2), 50 mM n-octyl-β-D-glucoside, 1 mM dithiothreitol, 20 mM NaF, 10 mM MgCl_2_, 1 μM CaCl_2_, 27 mol% PS, 5.0 mol% 1,2-dioleoyl-sn-glycerol (18:1/18:1-diacylglycerol) and 1 mM [γ-^32^P]ATP (100000 cpm/nmol). The reaction was initiated by adding the cell lysates, and it continued for 5 min at 30°C. Lipids were extracted from the mixture, and phosphatidic acid was separated by thin layer chromatography. The phosphatidic acid spot was scraped and counted by a liquid scintillation spectrophotometer.

### Statistical analysis

Statistical comparisons were performed using one-way ANOVA followed by a Tukey’s test.

## Results

### Cloning of full length DGKη3

**Fig 2:**
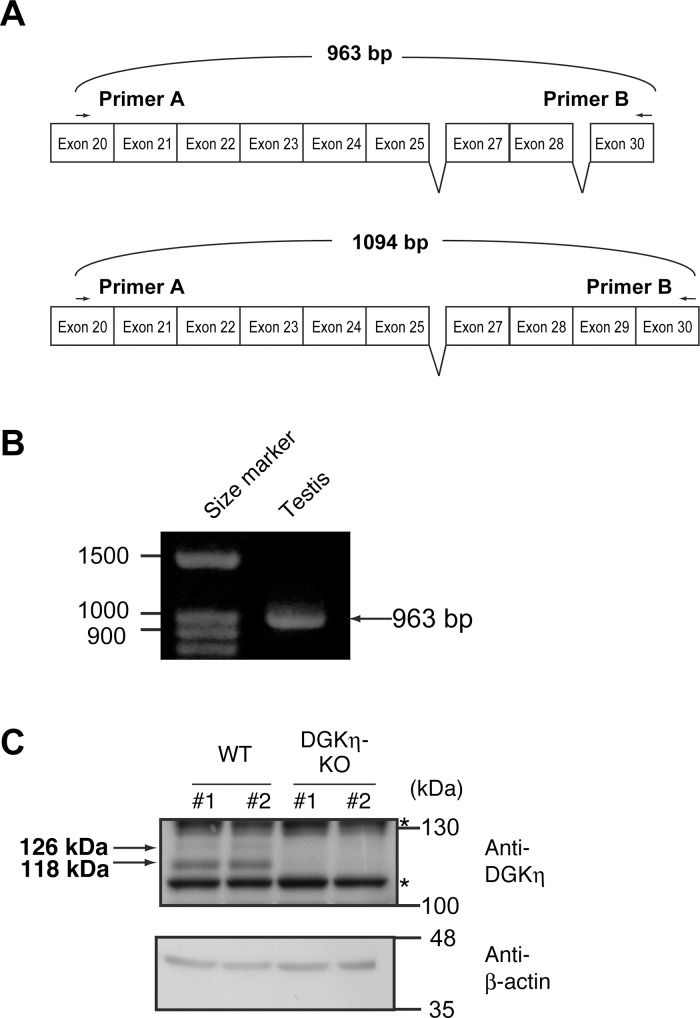
DGKη3 is derived from DGKη1. (A) Schematic representation of the primers used for RT-PCR. (B) RT-PCR analysis was carried out on mRNA prepared from mouse testis using primers A and B. Representatives of three independent experiments are shown. (C) The protein samples (50 μg) from the testes of 10-week-old wild-type (WT) and DGKη-knockout (KO) male mice were analyzed by Western blotting using anti-DGKη and β-actin antibodies. *: non-specific band.

We previously found a new alternative splicing product of DGKη gene, DGKη3, which lacks exon 26 encoding 31 amino acid residues [25] (Fig 1). However, it is still unknown whether the DGKη3 mRNA is a derivative of the DGKη1 mRNA or the DGKη2 mRNA. To clarify this, it is needed to determine whether DGKη3 gene contains exon 29, which produces DGKη2 (Figs 1 and 2A), or not. Thus, we performed RT-PCR using primer A (in exon 20) and B (in exon 30) indicated in Fig 2A. If DGKη3 contains exon 29, a 1094 bp product will be amplified (Fig 2A). If not, a 963 bp band will be detected (Fig 2A). As shown in Fig 2B, only the 963 bp product was amplified, indicating that the DGKη3 mRNA does not contain exon 29 and is derived from the DGKη1 mRNA. We next cloned and completely sequenced the full-length DGKη3 mRNA, and consequently confirmed that the DGKη3 mRNA is a derivative of the DGKη1 mRNA (Fig 1). Therefore, the protein product of DGKη3 does not contain a SAM domain (Fig 1).

Although the DGKη3 mRNA was strongly expressed in the testis, its protein band has not been detected in our previous report [25]. It is possible that the DGKη3 protein is unstable and quickly degraded. Therefore, in the present study, we added various protease inhibitors (see Experimental Procedures) into testis samples to prevent proteolysis, and performed Western blotting. As shown in Fig 2C, we robustly detected anti-DGKη antibody-reactive bands of 126 kDa, which approximately correspond to a calculated molecular mass of 124 kDa of DGKη3, and 118 kDa. Moreover, we confirmed that the 126 and 118 kDa bands were disappeared in testes of DGKη-knockout mice [24]. These results strongly suggest that the DGKη3 mRNA is translated in the testis. The lower band (118 kDa) may be a product of proteolytic degradation from the upper band (126 kDa).

### Identification of DGKη4

**Fig 3:**
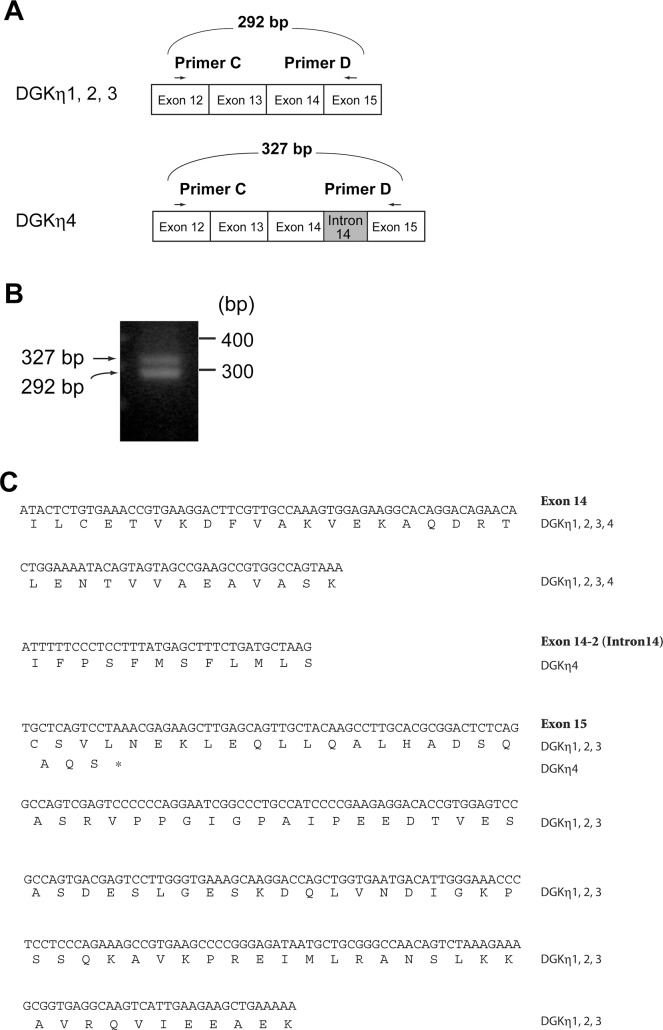
A new alternative splicing variant, DGKη4. (A) Schematic representation of the primers used for RT-PCR. (B) The nucleotide sequences and deduced amino acid sequences of DGKη1, DGKη2, DGKη3 and DGKη4 are shown. (C) RT-PCR analysis was carried out on mRNA prepared from mouse testis using primers C and D. Representatives of three independent experiments are shown.

When we performed RT-PCR for sequencing, we detected a longer DGKη mRNA (327 bp) that includes 35 bp of additional nucleotides derived from intron 14 (Fig 3A and 3B). This insertion leads to a frame shift, resulting in an in-frame stop codon in exon 15 and generating the extra 15 aa hydrophobic tail, IFPSFMSFLMSAQS (Fig 3A and 3C). Therefore, we designated it DGKη4 as a new splice variant of the DGKη gene (Fig 1). RT-PCR using primers C in exon 12 and primer D in exon 15 (Fig 3A and 3B) showed that the product derived from DGKη4 was approximately 46% and that derived from DGKη3 was approximately 54% (Fig 3B). The encoding protein of the DGKη4 gene stops at the middle of the intermediate region between separated catalytic subdomains (Fig 1). Therefore, the DGKη4 protein lacks the C-terminal half of the catalytic domain.

### Expression of DGKη3 and DGKη4 mRNAs

**Fig 4:**
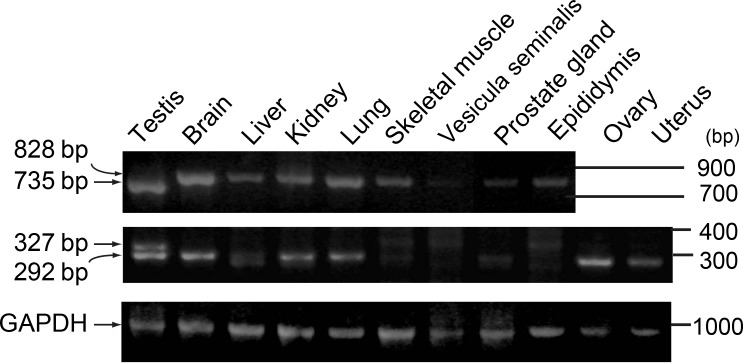
The mRNA expression of DGKη3 and η4 in mouse tissues. RT-PCR analysis was carried out on panels of cDNA from mouse normal tissues. Upper panel shows 828-bp and 735-bp cDNA fragments amplified using primers A and B. Center panel shows 292-bp and 327-bp cDNA fragments amplified using primers C and D. Lower panel exhibits a 978-bp cDNA fragment amplified for mouse glyceraldehyde phosphate dehydrogenase (GAPDH). Representatives of three independent experiments are shown.

In addition to the testis, expression of DGKη3 and η4 mRNAs in other organs such as brain, liver, kidney, lung, skeletal muscle, vesicula seminalis, prostate gland, epididymis, ovary and uterus were analyzed using primers A and B, which amplify 828 bp (DGKη1 and η2) and 735 bp (DGKη3) products, respectively, and were analyzed using primers C and D, which amplify 327 bp (DGKη1, η2 and η3) and 292 bp (DGKη4) products, respectively. Shionoya *et al*. reported that DGKη3 was not expressed in ovary or uterus whereas DGKη1 and η2 were expressed there. Thus, the expression of DGKη3 in brain, liver, kidney, lung, skeletal muscle, vesicula seminalis, prostate gland and epididymis was checked. We confirmed that the 735 bp band derived from DGKη3 mRNA was expressed in testis alone (Fig 4). On the other hand, the 828 bp product (DGKη1 and η2) was detected in brain, liver, kidney, lung, skeletal muscle, vesicula seminalis, prostate gland and epididymis.

The 327 bp product amplified from DGKη4 mRNA was also detected only in the testis (Fig 4). The 292 bp product (DGKη1, η2 and η3) was detected in testis, brain, liver, kidney, lung, skeletal muscle, prostate gland, ovary and uterus. In vesicula seminalis, epididymis, liver and skeletal muscle, other products having different lengths were detected, suggesting that other alternative splicing variants of the DGKη gene may exist. These results strongly suggest that DGKη3 and η4 mRNAs are testis-specifically expressed.

### DGK activities of DGKη3 and η4

**Fig 5:**
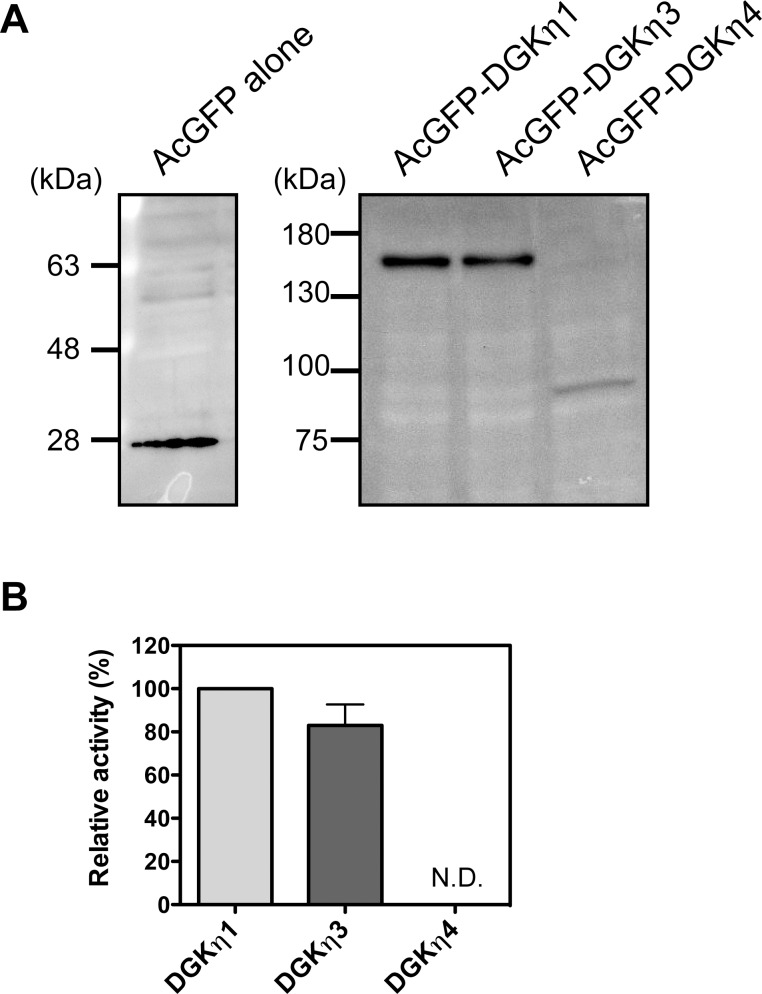
Catalytic activities of DGKη3 and η4. (A) Expression of AcGFP alone, AcGFP-DGKη1, η3 and η4 in COS-7 cells. COS-7 cells were transfected with pAcGFP vector alone, pAcGFP-DGKη1, η3 or η4. The cell lysates (12 μg of protein) were analyzed by Western blotting using anti-GFP antibody. (B) The relative activities of DGKη3 and η4 compared to DGKη1. The cell lysates (5 μg of protein/sample) were assayed for DGK activity (triplicate determinations). The background activities were subtracted and then the values were normalized for DGK expression levels visualized by Western blotting. The results are presented as the percentage of the value of DGKη1 and the mean ± S.D. of the values obtained in three separate experiments.

We next characterized properties of the newly identified splice variants, DGKη3 and η4. To measure DGK activities of protein products encoded by DGKη3 and η4 genes, these proteins were overexpressed in COS-7 cells. We confirmed expression of DGKη3, η4 and η1, which is a positive control (Fig 5A). Compared to DGK activity of DGKη1, DGKη3 exhibited almost the same (approximately 80%) activity (Fig 5B), indicating that DGKη3 is catalytically active. On the other hand, DGK activity of DGKη4 was not detectable (Fig 5B), indicating that this isoform is kinase negative.

### Subcellular localization of DGKη3 and η4 in NEC8 cells

**Fig 6:**
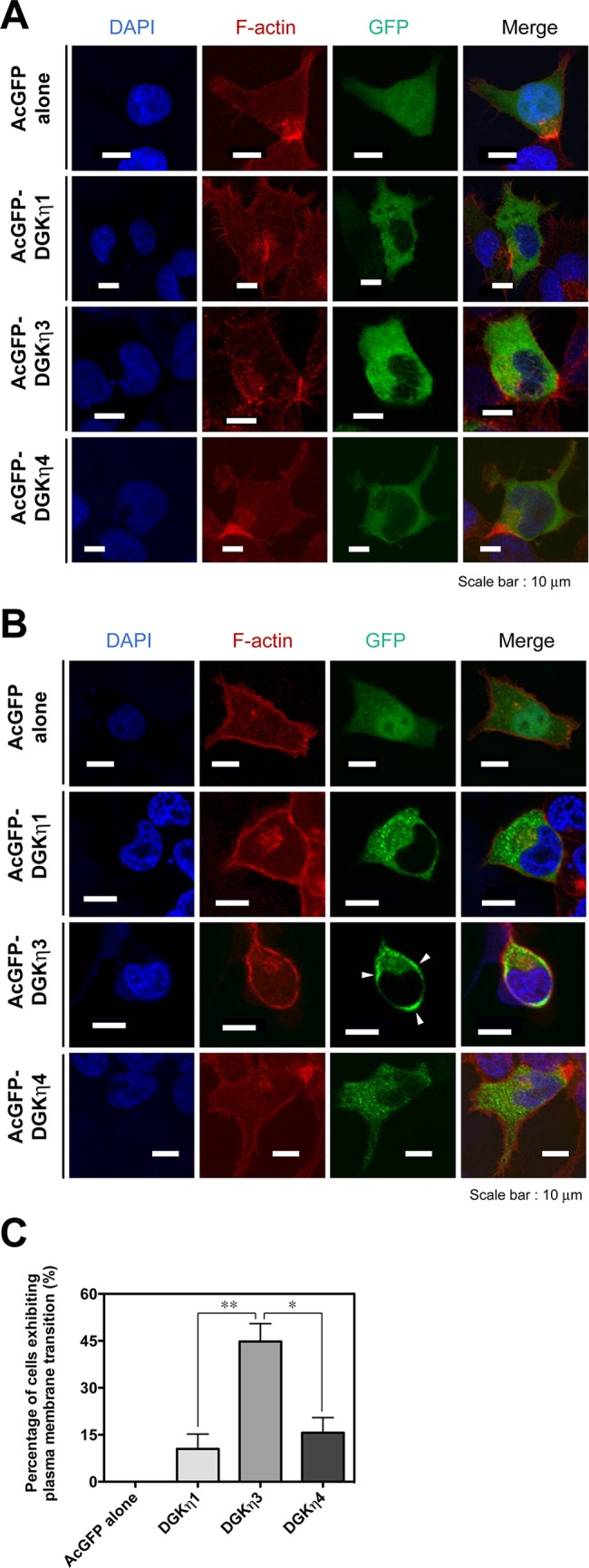
Subcellular localization of DGKη3 and η4 in NEC8 cells stimulated with osmotic stress. NEC8 cells were transfected with pAcGFP vector alone, pAcGFP-DGKη1, η3 or η4. After 24 h, the cells were serum starved for 3 h and incubated (A) 0 mM or (B) 500 mM sorbitol in RPMI-1640 for 30 min. The cells were fixed with 3.7% formaldehyde and then mounted onto coverslips. The fixed cells were stained using Alexa 594-conjugated phalloidin and 4’,6-diamino-2-phenyl indole (DAPI). Fluorescence images were obtained using an inverted confocal laser microscope. (C) The percentages of cells exhibiting translocation of AcGFP alone, AcGFP-DGKη1, η3 or η4 to the plasma membrane were scored. More than 20 cells expressing AcGFP alone, AcGFP-DGKη1, η3 or η4 were counted in each experiment. The results are the means ± S.D. of three separate experiments. *P<0.05, **P<0.01.

We next tested whether DGKη3 and η4 respond to stress stimulation. To address this, we determined subcellular localization of DGKη3 and η4 in NEC8 cells (human testicular germ cell tumor cell line) in the presence and absence of 0.5 M sorbitol (osmotic stress). AcGFP alone were broadly distributed in the cytoplasm and nucleus in the presence and absence of 0.5 M sorbitol (Fig 6A and 6B). In the absence of sorbitol, DGKη1, η3 and η4 were broadly distributed in the cytoplasm, and localization of either DGKη1, η3 or η4 at the plasma membrane was not detectable (Fig 6A). When osmotically shocked, DGKη1 and η4 were distributed in punctate vesicles in the cytoplasm (Fig 6B). Unexpectedly, DGKη3 was partly translocated to the plasma membrane and co-localized with actin cytoskeleton in an osmotic stress-dependent manner (Fig 6B). As shown in Fig 6C, DGKη3 was translocated to the plasma membrane in approximately 45% of the cells in response to osmotic stress, whereas DGKη1 and η4 were located at the plasma membrane in only 10–15% of the cells. These results strongly suggest that DGKη3 was specifically translocated to the plasma membrane in NEC8 cells in response to osmotic stress.

## Discussion

In this study, we cloned cDNA of the testis specific full length DGKη3 mRNA (Figs 1 and 2). Moreover, we found a new testis-specific alternative splicing product of the DGKη gene, DGKη4, during the cloning procedure (Figs 1 and 3). DGKη4 lacks half of the catalytic domain (Fig 1).

The DGKη3 mRNA is a splice variant derived from the DGKη1 mRNA but not from the DGKη2 mRNA (Figs 1 and 2). Therefore, DGKη3 does not contain the SAM domain (Fig 1). DGKη4 also lacks the SAM domain (Fig 1). In addition to DGKδ1 and δ2 [11, 14], DGKη2 formed an oligomer through its SAM domain [12]. However, it is unlikely that DGKη3 and η4 are able to form oligomers.

The biochemical and cell biological properties of DGKη3 and η4 were different from those of DGKη1. First, DGK activity was not detectable for DGKη4 (Fig 5). The loss of activity is reasonable because Rittiner *et al*. [26] revealed that a DGKη1 mutant lacking the C-terminal half of the catalytic domain did not show DGK activity. On the other hand, DGKη3 showed almost the same activity as DGKη1 (Fig 5), indicating that 31 amino acid-deletion between two coiled-coil structures (Fig 1) does not significantly affect its catalytic activity.

Second, the subcellular localization of DGKη3 was different from that of DGKη1 (Fig 6). Environmental stress causes testicular dysfunction [27, 28]. Thus, we tested whether DGKη3 and η4 respond to osmotic stress stimulation. DGKη1 and η2 were translocated to punctate vesicles in the cytoplasm in response to osmotic stress, as previously reported [12, 13, 26, 29]. In NEC8 cells DGKη1 or η4 were also osmotic stress-dependently translocated to punctate vesicles in the cytoplasm (Fig 5). Interestingly. DGKη3, but not DGKη1 or η4, was partly translocated to the plasma membrane and co-localized with F-actin in NEC8 cells in response to osmotic stress. Therefore, a 31 amino acid deletion between two coiled-coil structures in DGKη3 (Fig 1) would affect its osmotic stress-dependent localization. Thus, the length between two coiled-coil structures may be important for subcellular localization. The strong plasma membrane localization of DGKη3 was not observed in COS-7 cells (data not shown), suggesting that its translocation is cell line dependent.

It is possible that the splice variants DGKη3 and DGKη4 might be artifacts of cDNA preparation. However, this possibility is unlikely because the splice variants were detected only in the testis (Fig 4), although the artifacts would arise in every tissues. Moreover, we obtained the same RT-PCR products amplified from DGKη3 and DGKη4 mRNAs in three independent experiments. Furthermore, alternative splicing products of the DGKη gene (accession number XP_011243403, XP_011243404 and XP_006519232), which lack exon 26, are predicted and deposited into the NCBI database. These records are derived from a genomic sequence annotated using gene prediction method Gnomon, which is supported by mRNA and EST evidence. Finally, we robustly detected anti-DGKη antibody-reactive bands of 126 kDa, which approximately corresponds to a calculated molecular mass of 124 kDa of DGKη3, and 118 kDa (Fig 2C). Moreover, we confirmed that the 126 and 118 kDa bands were disappeared in testes of DGKη-knockout mice [24]. Because available anti-DGKη antibodies detect the C-terminal half of DGKη1–3, we are not able to detect endogenous DGKη4 protein at present. Thus, it is also possible that the DGKη4 mRNA is a non-coding RNA. However, this possibility is unlikely because the splice variant contains initiation methionine and stop codons. Even if the DGKη4 mRNA is a non-coding RNA, it is predicted to play specialized roles in the testis.

DGKη3 and η4 are expressed in the testis (Fig 4), especially in the secondary spermatocytes and round spermatids [25]. The round spermatids are generated from the secondary spermatocytes through the second meiotic division [30, 31], implying that these isoforms play specialized roles in meiosis during spermatogenesis. DGKη3 is catalytically active and stress-dependently localizes at the plasma membrane but not the cytoplasm where DGKη1 exists. DGKη4 exhibited the same localization with DGKη1 but is catalytically inactive. DGKη1 is known to enhance proliferation [18]. Thus, these isoforms may attenuate mitosis/proliferation and promote meiosis through their inactivity and different localization. Therefore, our present work would provide useful information to the study of spermatogenesis. However, to explore their specialized physiological functions, further study is required.

## Abbreviations

DGK: diacylglycerol kinase
KO: knockout

## References

[1] Goto K, Hozumi Y, Kondo H. Diacylglycerol, phosphatidic acid, and the converting enzyme, diacylglycerol kinase, in the nucleus. Biochim Biophys Acta. 2006;1761(5–6):535–41. Epub 2006/05/30. 10.1016/j.bbalip.2006.04.001 . 16731035

[2] Merida I, Avila-Flores A, Merino E. Diacylglycerol kinases: at the hub of cell signalling. Biochem J. 2008;409(1):1–18. Epub 2007/12/08. BJ20071040 [pii] 10.1042/BJ20071040 . 18062770

[3] Sakane F, Imai S, Kai M, Yasuda S, Kanoh H. Diacylglycerol kinases: why so many of them? Biochim Biophys Acta. 2007;1771(7):793–806. . 17512245 10.1016/j.bbalip.2007.04.006

[4] Sakane F, Imai S, Kai M, Yasuda S, Kanoh H. Diacylglycerol kinases as emerging potential drug targets for a variety of diseases. Curr Drug Targets. 2008;9(8):626–40. Epub 2008/08/12. . 18691010 10.2174/138945008785132394

[5] Topham MK. Signaling roles of diacylglycerol kinases. J Cell Biochem. 2006;97(3):474–84. . 16288460 10.1002/jcb.20704

[6] van Blitterswijk WJ, Houssa B. Properties and functions of diacylglycerol kinases. Cell Signal. 2000;12(9–10):595–605. 11080611 10.1016/s0898-6568(00)00113-3

[7] Sakai H, Sakane F. Recent progress on type II diacylglycerol kinases: the physiological functions of diacylglycerol kinase δ, η and κ and their involvement in disease. J Biochem. 2012;152(5):397–406. 10.1093/jb/mvs104 22984004

[8] Sakane F, Imai S, Kai M, Wada I, Kanoh H. Molecular cloning of a novel diacylglycerol kinase isozyme with a pleckstrin homology domain and a C-terminal tail similar to those of the EPH family of protein tyrosine kinase. J Biol Chem. 1996;271(14):8394–401. 8626538 10.1074/jbc.271.14.8394

[9] Klauck TM, Xu X, Mousseau B, Jaken S. Cloning and characterization of a glucocorticoid-induced diacylglycerol kinase. J Biol Chem. 1996;271(33):19781–8. 8702685 10.1074/jbc.271.33.19781

[10] Imai S, Kai M, Yasuda S, Kanoh H, Sakane F. Identification and characterization of a novel human type II diacylglycerol kinase, DGKκ. J Biol Chem. 2005;280(48):39870–81. . 16210324 10.1074/jbc.M500669200

[11] Sakane F, Imai S, Yamada K, Murakami T, Tsushima S, Kanoh H. Alternative splicing of the human diacylglycerol kinase δ gene generates two isoforms differing in their expression patterns and in regulatory functions. J Biol Chem. 2002;277(45):43519–26. 12200442 10.1074/jbc.M206895200

[12] Murakami T, Sakane F, Imai S, Houkin K, Kanoh H. Identification and characterization of two splice variants of human diacylglycerol kinase η. J Biol Chem. 2003;278(36):34364–72. . 12810723 10.1074/jbc.M301542200

[13] Kume A, Kawase K, Komenoi S, Usuki T, Takeshita E, Sakai H, et al The Pleckstrin Homology Domain of Diacylglycerol Kinase eta Strongly and Selectively Binds to Phosphatidylinositol 4,5-Bisphosphate. J Biol Chem. 2016;291(15):8150–61. Epub 2016/02/19. 10.1074/jbc.M115.648717 . 26887948 PMC4825017

[14] Harada BT, Knight MJ, Imai S, Qiao F, Ramachander R, Sawaya MR, et al Regulation of enzyme localization by polymerization: polymer formation by the SAM domain of diacylglycerol kinase δ1. Structure. 2008;16(3):380–7. Epub 2008/03/13. S0969-2126(08)00023-3 [pii] 10.1016/j.str.2007.12.017 . 18334213

[15] Imai S, Sakane F, Kanoh H. Phorbol ester-regulated oligomerization of diacylglycerol kinase δ linked to its phosphorylation and translocation. J Biol Chem. 2002;277(38):35323–32. 12084710 10.1074/jbc.M202035200

[16] Knight MJ, Joubert MK, Plotkowski ML, Kropat J, Gingery M, Sakane F, et al Zinc Binding Drives Sheet Formation by the SAM Domain of Diacylglycerol Kinase δ. Biochemistry. 2010;49(44):9667–76. Epub 2010/09/23. 10.1021/bi101261x . 20857926 PMC3035719

[17] Komenoi S, Takemura F, Sakai H, Sakane F. Diacylglycerol kinase eta1 is a high affinity isozyme for diacylglycerol. FEBS Lett. 2015;589(11):1272–7. Epub 2015/04/12. 10.1016/j.febslet.2015.03.032 . 25862496

[18] Yasuda S, Kai M, Imai S, Takeishi K, Taketomi A, Toyota M, et al Diacylglycerol kinase η augments C-Raf activity and B-Raf/C-Raf heterodimerization. J Biol Chem. 2009;284(43):29559–70. Epub 2009/08/28. M109.043604 [pii] 10.1074/jbc.M109.043604 19710016 PMC2785589

[19] Nakano T, Iravani A, Kim M, Hozumi Y, Lohse M, Reichert E, et al Diacylglycerol kinase eta modulates oncogenic properties of lung cancer cells. Clinical & translational oncology: official publication of the Federation of Spanish Oncology Societies and of the National Cancer Institute of Mexico. 2014;16(1):29–35. Epub 2013/04/11. 10.1007/s12094-013-1036-y 23572183 PMC3883989

[20] Usuki T, Sakai H, Shionoya T, Sato N, Sakane F. Expression and localization of type II diacylglycerol kinase isozymes delta and eta in the developing mouse brain. The journal of histochemistry and cytochemistry: official journal of the Histochemistry Society. 2015;63(1):57–68. Epub 2014/11/02. 10.1369/0022155414559130 . 25362140 PMC4395997

[21] Baum AE, Akula N, Cabanero M, Cardona I, Corona W, Klemens B, et al A genome-wide association study implicates diacylglycerol kinase eta (DGKH) and several other genes in the etiology of bipolar disorder. Mol Psychiatry. 2008;13(2):197–207. Epub 2007/05/09. 10.1038/sj.mp.4002012 17486107 PMC2527618

[22] Weber H, Kittel-Schneider S, Gessner A, Domschke K, Neuner M, Jacob CP, et al Cross-disorder analysis of bipolar risk genes: further evidence of DGKH as a risk gene for bipolar disorder, but also unipolar depression and adult ADHD. Neuropsychopharmacology: official publication of the American College of Neuropsychopharmacology. 2011;36(10):2076–85. 10.1038/npp.2011.98 21654738 PMC3158324

[23] Zeng Z, Wang T, Li T, Li Y, Chen P, Zhao Q, et al Common SNPs and haplotypes in DGKH are associated with bipolar disorder and schizophrenia in the Chinese Han population. Mol Psychiatry. 2011;16(5):473–5. Epub 2010/08/25. 10.1038/mp.2010.86 . 20733578

[24] Isozaki T, Komenoi S, Lu Q, Usuki T, Tomokata S, Matsutomo D, et al Deficiency of diacylglycerol kinase eta induces lithium-sensitive mania-like behavior. J Neurochem. 2016;138(3):448–56. Epub 2016/05/12. 10.1111/jnc.13661 . 27167678

[25] Shionoya T, Usuki T, Komenoi S, Isozaki T, Sakai H, Sakane F. Distinct Expression and Localization of the Type II Diacylglycerol Kinase Isozymes δ, η and κ in the Mouse Reproductive Organs. BMC Dev Biol. 2015;15(1):6. 25613821 10.1186/s12861-015-0055-z PMC4308931

[26] Rittiner JE, Brings VE, Zylka MJ. Overexpression of diacylglycerol kinase eta enhances Galphaq-coupled G protein-coupled receptor signaling. Mol Pharmacol. 2014;85(5):800–10. Epub 2014/03/13. 10.1124/mol.113.091280 24608858 PMC3990018

[27] Agarwal A, Desai NR, Ruffoli R, Carpi A. Lifestyle and testicular dysfunction: a brief update. Biomedicine & pharmacotherapy = Biomedecine & pharmacotherapie. 2008;62(8):550–3. Epub 2008/09/06. 10.1016/j.biopha.2008.07.052 . 18771892

[28] Sharpe RM. Lifestyle and environmental contribution to male infertility. British medical bulletin. 2000;56(3):630–42. Epub 2001/03/20. . 11255550 10.1258/0007142001903436

[29] Matsutomo D, Isozaki T, Sakai H, Sakane F. Osmotic shock-dependent redistribution of diacylglycerol kinase η1 to non-ionic detergent-resistant membrane via pleckstrin homology and C1 domains. J Biochem. 2013;153(2):179–90. 10.1093/jb/mvs130 23127959

[30] Cheng YH, Wong EW, Cheng CY. Cancer/testis (CT) antigens, carcinogenesis and spermatogenesis. Spermatogenesis. 2011;1(3):209–20. 10.4161/spmg.1.3.17990 22319669 PMC3271663

[31] Cooke HJ, Saunders PT. Mouse models of male infertility. Nature reviews Genetics. 2002;3(10):790–801. 10.1038/nrg911 . 12360237

